# Young children share more under time pressure than after a delay

**DOI:** 10.1371/journal.pone.0248121

**Published:** 2021-03-16

**Authors:** Maria Plötner, Robert Hepach, Harriet Over, Malinda Carpenter, Michael Tomasello

**Affiliations:** 1 Department of Developmental and Comparative Psychology, Max Planck Institute for Evolutionary Anthropology, Leipzig, Germany; 2 Department of Clinical Child and Adolescent Psychology, Leipzig University, Leipzig, Germany; 3 Department of Experimental Psychology, University of Oxford, Oxford, United Kingdom; 4 Department of Psychology, University of York, York, United Kingdom; 5 School of Psychology & Neuroscience, University of St Andrews, St Andrews, United Kingdom; 6 Department of Psychology and Neuroscience, Duke University, Durham, NC, United States of America; Jiangsu Normal University, CHINA

## Abstract

Adults under time pressure share with others generously, but with more time they act more selfishly. In the current study, we investigated whether young children already operate in this same way, and, if so, whether this changes over the preschool and early school age years. We tested 144 children in three age groups (3-, 5-, and 7-year olds) in a one-shot dictator game: Children were given nine stickers and had the possibility to share stickers with another child who was absent. Children in the Time Pressure condition were instructed to share quickly, whereas children in the Delay condition were instructed to take time and consider their decision carefully. Across ages, children in the Time Pressure condition shared significantly more stickers than children in the Delay condition. Moreover, the longer children waited, the less they shared. Thus, children, like adults, are more prosocial when acting spontaneously than after considering their decision more carefully.

## Introduction

Young children are highly prosocial (for an overview, see, e.g., [1]). From early in ontogeny, children provide others with information and instrumental help [2–4], comfort others who are in distress [5, 6], and share resources with others [7].

There has been a debate about whether prosociality stems from intuitive or reflective tendencies [8]. A recent study with toddlers has found that the speed of helping was correlated with a greater frequency of helping, suggesting that prosocial behavior indeed seems to be governed by intuitive processes [9]. However, helping and sharing are two distinct types of prosocial behavior varying in onset, course of development and underlying mechanisms and should thus be considered separately [5, 10, 11]. Jensen, Vaish, and Schmidt [12] and others have noted that the act of sharing resources with others is remarkable in itself, since “this is not the rational, self-interested thing to do” (p. 2). Indeed, prosocial behaviors such as sharing are prone to being exploited by selfish others [13]. Recent work using economic games with adults suggests that deciding whether one’s contributions might be subject to exploitation by others requires deliberation. In one study, participants in a public goods game who were asked to contribute under time pressure gave more to a common pool of resources than participants who were asked to delay their decision and consider it carefully [14]. In addition, decision time was negatively correlated with participants’ contributions: The longer participants took to decide, the less they contributed to the common good. The speed with which participants reach their decision is indicative of how spontaneous their decisions were. While fast reactions are automatic and likely based on intuition, slow reactions are more likely to involve deliberate processing [15]. Rand and colleagues’ work has been interpreted as evidence that humans are intuitively prosocial while deliberation undermines prosocial sharing [16, see also 17]. There has been considerable debate about these findings since then. While some empirical studies challenge Rand and colleagues’ findings [18, 19], a meta-analysis on 67 studies supports the initial effect [20].

However, most studies on the topic have been done with adults who have undergone a long period of internalizing societal norms and values. Developmental studies are essential to better understand the foundations of prosocial tendencies [21]. While very young children share rather indiscriminately, sharing becomes more selective and flexible with age [22]: Children start considering merit [23], reciprocity [24], friendship [25], and group membership in their sharing behavior [26]. This might indicate that children start off by sharing with others readily, and with age think more about who is a worthy recipient [1, 22]. However, there are also studies suggesting that children have to overcome selfishness with effort, indicative of a reflective prosociality [see also 27]. In a study by Aguilar-Pardo, Martínez-Arias, and Colmenares [28], 4- to 6-year-old children who shared altruistically in a one-shot dictator game performed better in an inhibitory control task than non-altruists. Aguilar-Pardo et al. suggested that children’s self-maximizing is a natural tendency that needs to be inhibited in order to facilitate costly sharing. Similar results have been found with older children [29–31].

However, correlations between inhibitory processes and sharing are not a direct test of the effect of reflection on children’s generosity. Only a direct manipulation of reflective processes is suitable to test for this effect [32]. There are no previous studies to our knowledge that investigate the influence of deliberation on prosocial tendencies in children using an experimental design. In the current study, we used a highly simplified version of Rand and colleagues’ [14] task. We tested 144 children in three age groups (3-, 5-, and 7-year-olds) in a one-shot dictator game. Children were given nine stickers and had the possibility to share stickers with another child who was absent. Children in the Time Pressure condition were instructed to share quickly whereas children in the Delay condition were instructed to wait and consider their decision carefully before sharing. We hypothesized that children would share more under time pressure and less after a delay and that decision time would be negatively correlated to the number of stickers shared.

## Method

### Ethics statement

The present study strictly adhered to the legal requirements of the country in which it was conducted, and a detailed procedure was approved in advance by the Max Planck Institute for Evolutionary Anthropology Human Subjects Committee. In addition, parents of all children who participated in the study gave informed written consent.

### Participants

Participants were 144 children in 3 age groups. Forty-eight children were 3 years old (mean age: 3 years, 5 months; age range: 3 years, 2 months to 3 years, 7 months), 48 were 5 years old (mean age: 5 years, 5 months; age range: 5 years, 3 months to 5 years, 8 months) and 48 were 7 years old (mean age: 7 years, 5 months; age range: 7 years, 2 months to 7 years, 8 months). In each age group and condition, half of the participants were female and half were male (*n* = 12 girls and *n* = 12 boys per age group and condition). Children were recruited through a database of parents who had agreed to participate in studies on child development. The sample size was specified prior to data collection, based on typical sample sizes in this field.

Forty-two additional children were tested but excluded from analysis for video-camera error (*n*_3-year-olds_ = 2), experimenter error (e.g., giving incorrect or incomplete instructions or presenting the incorrect number of stickers) (*n*_3-year-olds_ = 6; *n*_5-year-olds_ = 6; *n*_7-year-olds_ = 1), because the child began sharing stickers before the experimenter finished the instructions (*n*_5-year-olds_ = 3; *n*_5-year-olds_ = 3), or because the child did not correctly answer the control question (see below; *n*_3-year-olds_ = 19; *n*_5-year-olds_ = 2) (Many of these 3-year-olds failed the control question because they said that the stickers they had shared were for their mother, or a friend, or for themselves later. This may have been because the sharing situation with an absent recipient was rather abstract for 3-year-olds).

### Materials and design

Materials were two identical orange place mats, nine identical, colorful star-shaped stickers and a photograph of a recipient child who was gender-matched to participants.

Children were randomly assigned to one of two experimental conditions.

### Setup and procedure

**Fig 1:**
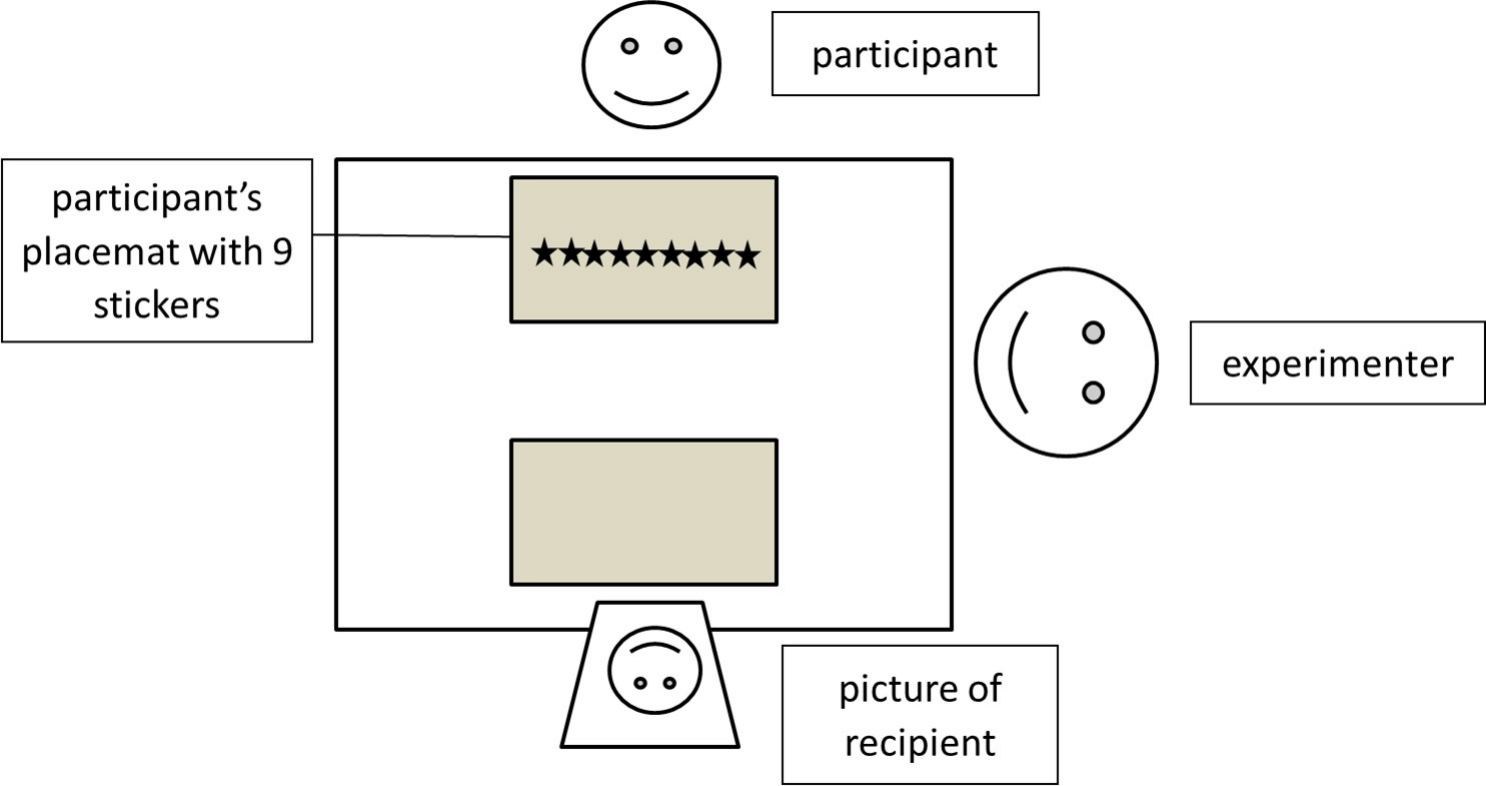
Set-up of the study. The gender of the recipient child on the picture matched that of the participant.

Testing was conducted in a quiet room in local nurseries or after-school care. Children sat at a table with the experimenter (Fig 1). The experimenter placed the two place mats on the table and told participants that one was for them. He then put the photograph of the recipient on the second place mat, saying, “And this place mat is for Lukas/Sarah. Look, this is a picture of Lukas/Sarah. This is a boy/girl from another kindergarten and he/she is the same age as you”. Then the experimenter brought out a stack of stickers and said, “Here I have so many star stickers. They are all for you! Let’s count how many there are.” After counting the nine stickers and helping children place them in a row in front of them, the experimenter said, “Unfortunately Lukas/Sarah could not come today, and he/she could not get any stickers. But if you want to, you can give some of your stickers to Lukas/Sarah. You can give some, but you don’t have to. As you like. The stickers you want to take home you can keep on the place mat. The stickers you want to give to Lukas/Sarah you can place on his/her place mat.” To check whether children understood these instructions, they were asked to repeat where to put the stickers they wanted to keep and where to put those they wanted to share. If participants failed to do this correctly, the instructions were repeated.

In the Time Pressure condition, the experimenter then said, “You have to hurry, you don’t have much time. Quick, you can start, you can give as many as you want now.” In the Delay condition, in contrast, the experimenter said, “Do it slowly, you have plenty of time. Think carefully about how many you want to give, then you can start and give as many as you want.” The experimenter then asked children to tell him when they were finished and turned away as if he was writing something down. If he noticed that children had finished allocating the stickers but did not say anything for 10 seconds, the experimenter asked, “Are you done?” If children said “Yes,” the procedure was finished; if children said, “No,” the instructions were repeated. To make sure that children had understood the procedure, the experimenter asked a control question at the very end: “Who gets the stickers which are here now?” (pointing to the stickers of the participant) and “Who gets the stickers which are here now?” (pointing to the stickers of the recipient child).

### Coding and reliability

The number of stickers that were shared with the recipient was coded from videotape. Reliability coding of the full sample by a naive coder who was unaware of the hypotheses revealed a very good agreement on the number of stickers shared (*ICC* = 0.95). We also coded the latency of sharing the first sticker (or, if children shared no stickers, the latency of saying they were done) from the moment that the experimenter’s instruction was finished. Reliability coding of the full sample by a naive coder who was unaware of the hypotheses revealed an acceptable agreement on sharing latency (*ICC* = 0.78).

### Statistical analyses

As a manipulation check, we tested whether there was a significant difference in sharing latency between conditions. As latency did not follow a normal distribution in the two conditions we used a Mann-Whitney *U* test.

The main analyses were run in *R* (version 3.3.1; [33]) using the function glmer (package lme4; version 1.1–17; [34]). The number of stickers shared was a count response with a lower (0) and an upper limit (9). We therefore analyzed the data using a Generalized Linear Mixed Model (GLMM, [35]) with binomial error structure and repeated observations on the proportions of stickers shared. Condition, age, and their interaction were included as fixed effects into the model. Child identity was included as a random intercept and gender was included as a control variable. We assessed model stability by comparing the estimates obtained from a model based on all data to those obtained from models with the levels of the random effects excluded one at a time. This revealed no issues of model stability (S1 Table). Further analyses indicated no issue of overdispersion (parameter = 0.68, *X*^2^ = 94.31, *df* = 139, *p* = .99).

To test the statistical significance of the main predictor variables of interest (condition, age, and their interaction) we first conducted an omnibus test by comparing the fit of the full model with that of a reduced model comprising only gender and the random intercept using a likelihood ratio test [36]. To test the significance of the interaction of age and condition, we compared the fit of the full model to that of a reduced model without the interaction. Tests of the main effects were derived using likelihood ratio tests comparing the fit of a model with the predictor of interest to that of a model without the predictor [37].

To investigate the correlation between latency to share and sharing, we conducted a Pearson correlation on the log-transformed sharing latency and the number of stickers shared.

## Results

### Manipulation check

Children in the Time Pressure condition showed a shorter sharing latency (*Mdn*: 3 seconds, *range*: 0 to 105 seconds) than did children in the Delay condition (*Mdn* = 16 seconds, *range* = 0 to 150 seconds; Mann-Whitney *U*(*n*_1_ = *n*_2_ = 71) = 532.5, *p* < .001). This effect remained when excluding children who shared zero stickers from the analysis.

### Main analyses

The omnibus statistical test of the predictor variables of interest revealed an improved model fit compared to the reduced model lacking the predictors age, condition, and their interaction (*X*^2^ = 18.77, *df* = 3, *p* < .001). There was no interaction of condition and age on the number of stickers children shared (*X*^2^ = 0.04, *df* = 1, *p* = .84) (S1 Fig).

**Fig 2:**
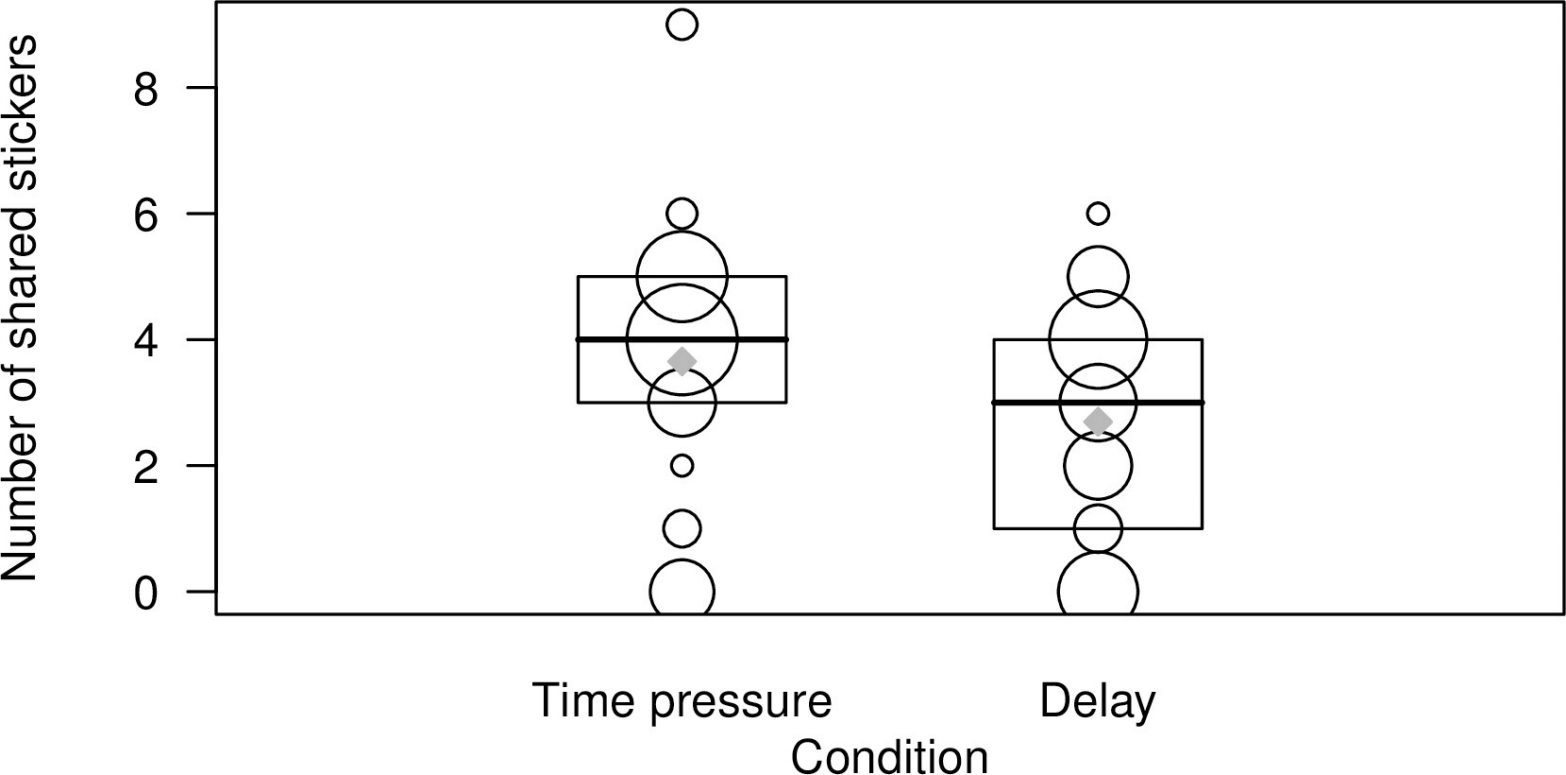
Depiction of the main effect of condition on the number of stickers shared. Bigger bubbles represent higher numbers of participants. Lines represent medians, boxes represent quartiles, diamonds represent means.

However, there was a main effect of condition: Children shared more stickers in the Time Pressure (*M* = 3.65, *SD* = 1.89) compared to the Delay (*M* = 2.69, *SD* = 1.73) condition (*X*^2^ = 10.27, *df* = 1, *p* = .001) (Fig 2). An exploratory analysis on the equality of variances revealed that there was no difference in the variance of sharing between conditions (*F*(71, 71) = 0.84, *p* = .46).

In addition, there was a main effect of age: Seven-year-olds (*M* = 3.5, *SD* = 1.4) and 5-year-olds (*M* = 3.54, *SD* = 1.56) shared more stickers than 3-year-olds (*M* = 2.48, *SD* = 2.33) (*X*^2^ = 8.88, *df* = 1, *p* = .003). There was no effect of gender on children’s sharing (*X*^2^ = 0.25, *df* = 1, *p* = .62).

**Fig 3:**
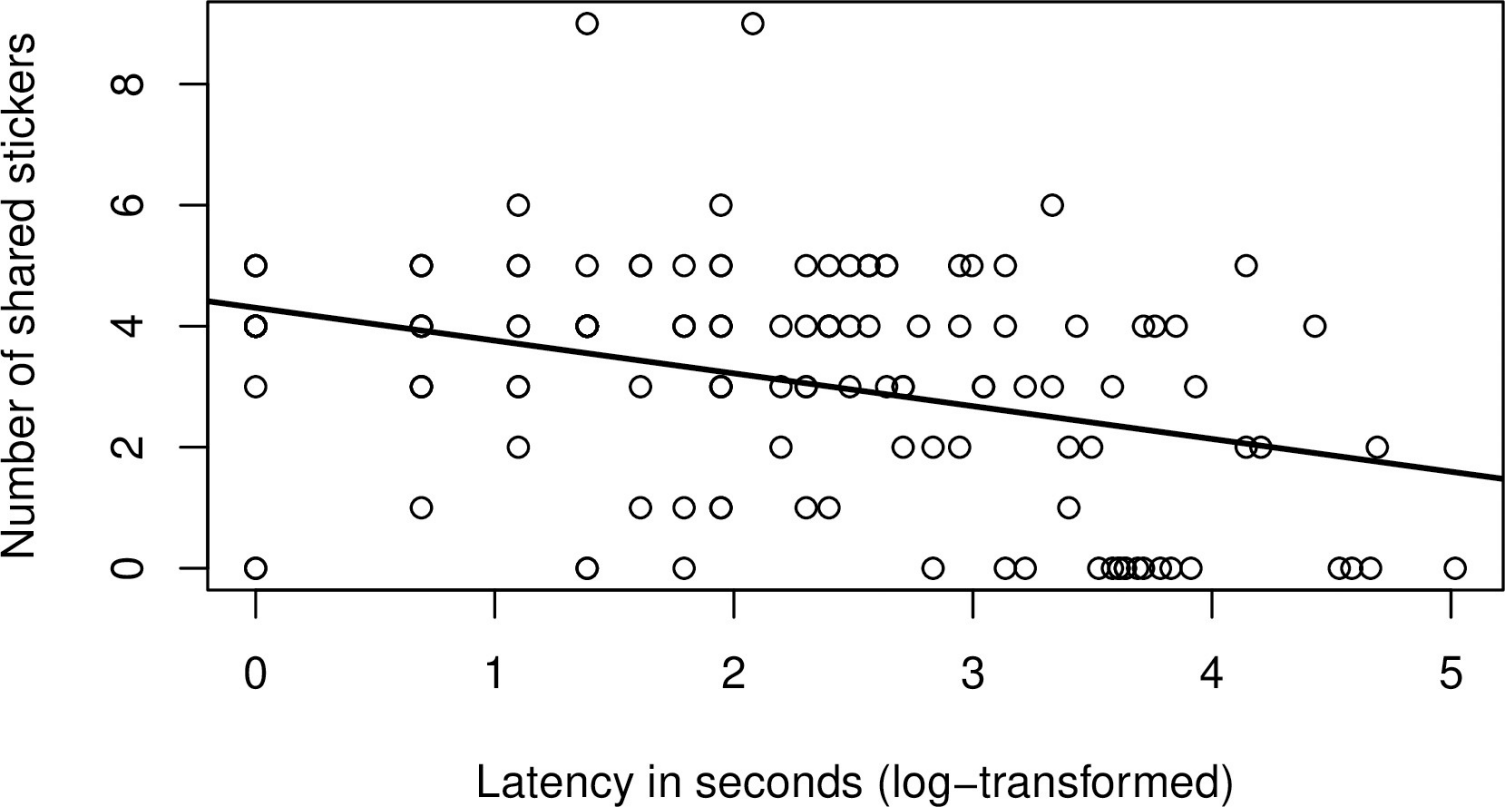
Depiction of the correlation between the log-transformed latency to share stickers and the number of stickers shared.

The correlational analysis between the log-transformed sharing latency and the number of stickers shared revealed a medium-sized negative correlation (Pearson’s *r*(142) = -.38, *p* < .001) (Fig 3).

## Discussion

This study investigated the effect of time pressure versus delay on 3-, 5-, and 7-year-old children’s willingness to share stickers with an absent recipient in a one-shot dictator game. We were interested in whether children share more under time pressure than when instructed to wait and think carefully about their decision. The manipulation check revealed that, as intended, children took more time before sharing in the Delay condition compared to the Time Pressure condition. We also found that younger children shared fewer stickers than older children. This is in line with previous research, which has found that children become more generous with age [38, 39]. More importantly, we found a main effect of condition in the hypothesized direction: Children from 3 years of age shared significantly more stickers in the Time Pressure condition than children in the Delay condition. In addition, sharing latency was negatively correlated to the number of stickers shared: The longer children waited before sharing, the less they gave to the recipient. This indicates that when acting spontaneously, children are more generous than after considering their decision carefully.

These findings are in line with Rand and colleagues’ work on adults [14, 16] and suggest an intuitive tendency for prosociality [8, 9], as intuitive responses promoting more generous sharing seem to be undermined by the instruction to wait and deliberate. In light of previous findings showing that cognitive control enhances children’s sharing [29, 30], our results might seem unexpected at first glance. Studies have found that children’s spontaneous sharing is less generous than their fairness norms would imply [40] and inhibition is suggested to help children successfully act upon these norms [29]. However, prosocial behavior can be driven by different motivations: a genuine concern for others or self-interested strategic concerns. Children in the age range we tested are capable of both. For example, studies show that even toddlers are intrinsically motivated to be prosocial [41] and preschoolers’ sharing can be driven by an empathic concern for others [42]. However, preschoolers also share strategically sometimes, especially if they can expect reciprocity [43, 44]. Genuine and strategic prosocial acts seem to have different underlying mechanisms [44]. One possibility is that behavioral inhibition might foster strategic sharing specifically in situations in which, for example, reciprocation is possible. In contrast, sharing based on a genuine concern for others, i.e. sharing without the prospect of future reciprocation (e.g., because the recipient is absent, as in our study), might be an intuitive tendency that might be undermined by reflection. Indeed a meta-analysis on adults shows that reflection appears to undermine altruistic sharing only, but not strategic sharing [20].

It is also possible that children used a different method of sharing in each condition. Work by Sommerville and colleagues has shown that even infants have a preference for equal distributions [45, 46]. On the other hand, older children’s sharing behavior has been found to be linked with cognitive abilities such as number cognition [47]. Hypothetically, children might have defaulted to an equality heuristic when under time pressure, and grabbed an amount that approximated half the stickers. In contrast, they might have used a more deliberate counting strategy when given a time delay, resulting in more limited sharing.

We do not yet know what is driving this effect in either adults or children. Since this is the first study using this kind of manipulation in young children, more research is needed to explore underlying mechanisms. There are two potential explanations for the effect. Rand and colleagues argued that adults’ intuitive tendency for prosociality is most likely based on social learning. If one grows up in a world in which being prosocial is the most effective outcome in the long run, one will internalize this tendency and thus intuitively act prosocially [16]. Since some scholars suggest that socialization processes shape the development of prosocial behaviors from birth [48], one could argue that 3 years of socialization is enough to result in an intuitive prosocial tendency. An alternative interpretation is that intuitive prosociality could be a natural, innate tendency of humans grounded in their evolutionary history [49]. This tendency could have evolved because humans were highly interdependent and thus had a high interest in the well-being of others, resulting in the evolution of specific skills and motivations to support prosocial action [49, 50]. Either way, our findings suggest that by 3 years of age young children already display an intuitive prosocial tendency.

In general, infants and very young children are often immediately prosocial, but with age, they begin to take into account other factors, including whether their partner is a potential reciprocator or cheater [1, 22]. The current study shows that when asked to take time to reflect upon their decision before sharing, children already from 3 years of age potentially begin to consider alternative, more self-interested strategies. Prosociality thus seems to be a deeply-rooted intuitive tendency already in early childhood that can be undermined by reflection.

## Supporting information

S1 Fig Depiction of the effect of age and condition on the number of shared stickers. Bigger bubbles represent higher numbers of participants. Lines represent medians, boxes represent quartiles, diamonds represent means.
(TIF)

S1 Table Model stability. Original, minimum and maximum model estimates obtained from comparisons of a model based on all data to those obtained from models with the levels of the random effects excluded one at a time.
(DOCX)

S1 Data (XLSX)
