# Supplementary figures and images for: Young children share more under time pressure than after a delay
Source: PLoS One. 2021 Mar 16;16(3):e0248121. doi: 10.1371/journal.pone.0248121 (PMC7963052; doi:10.1371/journal.pone.0248121)

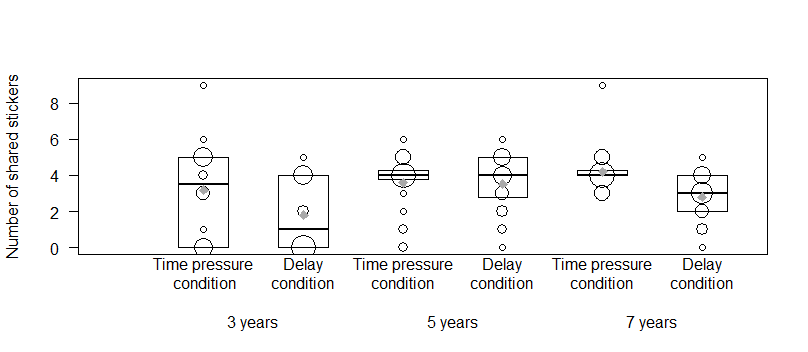

Supplement: S1 Fig — Bigger bubbles represent higher numbers of participants. Lines represent medians, boxes represent quartiles, diamonds represent means. (TIF) [file pone.0248121.s001.tif]
