# Supplementary material for: Young children share more under time pressure than after a delay
Source: PLoS One. 2021 Mar 16;16(3):e0248121. doi: 10.1371/journal.pone.0248121 (PMC7963052; doi:10.1371/journal.pone.0248121)
Supplement: S1 Table — Original, minimum and maximum model estimates obtained from comparisons of a model based on all data to those obtained from models with the levels of the random effects excluded one at a time. (DOCX) [file pone.0248121.s002.docx]

**S1 Table. Model stability.**

|  | **Original estimate** | **Minimum estimate** | **Maximum estimate** |
| --- | --- | --- | --- |
| **Intercept** | -1.75 | -1.89 | -1.62 |
| **Condition^a^** | 0.62 | 0.38 | 0.75 |
| **Age** | 0.16 | 0.14 | 0.17 |
| **Gender^b^** | -0.08 | -0.12 | -0.04 |
| **Condition^a^*Age** | -0.02 | -0.04 | 0.01 |
| **Child identity** | 0.59 | 0.53 | 0.6 |

Original, minimum and maximum model estimates obtained from comparisons of a model based on all data to those obtained from models with the levels of the random effects excluded one at a time.

^a^ condition = time pressure

^b^ gender = female
